# Cost-effectiveness of injury prevention - a systematic review of municipality based interventions

**DOI:** 10.1186/1478-7547-8-17

**Published:** 2010-09-10

**Authors:** Harald Gyllensvärd

**Affiliations:** 1 Department of Medical and Health Sciences, Linköping University, SE-581 83 Linköping, Sweden

## Abstract

**Background:**

Injuries are a major cause of mortality and morbidity which together result in avoidable societal costs. Due to limited resources, injury prevention interventions need to demonstrate cost-effectiveness to justify their implementation. However, the existing knowledge in this area is limited. Consequently, a systematic review is needed to support decision-making and to assist in the targeting of future research. The aim of this review is to critically appraise the published economic evidence of injury prevention interventions at the municipal level.

**Methods:**

A search strategy was developed to focus a literature search in PubMed, Embase, Cochrane and NHS EED. Studies were eligible for inclusion if they were economic evaluations of injury prevention interventions that could be implemented by municipalities; had a relevant comparison group; did not include any form of medication or drug use; and were assessed as having at least an acceptable quality from an economic point of view. Articles were screened in three steps. In the final step, studies were critically appraised using a check-list based on Drummond's check-list for assessing economic evaluations.

**Results:**

Of 791 potential articles 20 were accepted for inclusion. Seven studies showed net savings; four showed a cost per health score gained; six showed both savings and a cost per health score gained but for different time horizons and populations; and three showed no effect. The interventions targeted a range of areas such as traffic safety, fire safety, hip fractures, and sport injuries. One studied a multi-targeted community-based program. Only six articles used effectiveness data generated within the study.

**Conclusions:**

The results indicate that there are injury prevention interventions that offer good use of societal resources. However, there is a lack of economic evidence surrounding injury prevention interventions. This lack of evidence needs to be met by further research about the economic aspects of injury prevention interventions to improve the information available for decision-making.

## Background

Injuries are a major cause of morbidity and mortality and result in great costs to society. Annually more than five million people worldwide are killed due to injuries, which account for 9% of global mortality. Even more people are temporary or permanent disabled [1]. It is also estimated that injuries account for 14% of global life years lost when using the measure Years of Life Lost [2]. Injury prevention interventions could mitigate the impact of injuries on health, and at the moment a research project is running to explore what the success factors are for municipality based injury preventative and safety promotion work [3]. Here, a municipality is defined as an administrative entity for local governance in a defined territory. As a part of the mentioned research project the importance of achieving cost-effectiveness is recognized. This is important because it is insufficient for interventions to demonstrate effectiveness only to justify implementation due to limited resources. This calls into question whether injury prevention interventions can be seen as cost-effective or not; that is, whether or not they can be seen as good use of societal resources.

Systematic reviews of the cost-effectiveness of different interventions provide excellent overviews of the current state of knowledge about various topics. They can facilitate decision-making by providing compiled and critically appraised evidence on the cost-effectiveness of different interventions. This is especially warranted in a world where costs are increasing and budgets become more financially constrained. In the end of the day decision-makers need to make and justify decisions on how to allocate societal resources so that maximum health outcome is obtained for a confined budget.

Previously, some reviews targeting the cost-effectiveness of different injury prevention interventions have been conducted. For instance, Smith and Fordham [4] reviewed the cost-effectiveness of "interventions in reducing the risk of new falls, or modifying the harm caused in the event of a fall, for the general unselected population of the elderly living independently in the community". Also, Miller and Levy [5] reviewed cost-outcome analyses in injury prevention and control, and made estimates from the literature found. However, there is a paucity of economic evidence concerning prevention in general [6] and in injury prevention as well. Hence, it is important to compile the existing evidence and reveal important gaps for future research to target.

This systematic review of published literature was conducted to elucidate what options are available for a decision-maker, at the municipal level, searching economic evidence on injury prevention interventions. Therefore, the objective of this review study was to systematically identify, critically appraise, and compile economic evaluations of injury prevention interventions that could be conducted by municipalities.

## Methods

### Search strategy

A search strategy was developed in collaboration with a librarian. The strategy comprised four different parts: (a) injuries or accidents, (b) prevention, (c) economic evaluation, and (d) demarcation. MeSH terms such as accident prevention, wounds and injuries, prevention and control, primary prevention, protective devices, and cost-benefit analysis were used in different combinations in all databases except in Embase; in Embase similar terms were identified and used by utilising the thesaurus.

Searches were conducted in PubMed, Embase, Cochrane, and NHS EED and they were limited to the last ten years, from 1998 until the beginning of August 2008. Additional searches were also conducted to include the not yet MeSH indexed articles. The searches used the MeSH terms' entry terms and were limited to 2008 or the last 180 days. The search strategies used are available - see additional file 1 and 2.

### Inclusion criteria

For inclusion, the study should

• be an economic evaluation of an injury prevention intervention (cost-effectiveness, cost-benefit, or cost-utility analysis);

• include some sort of comparison (randomised controlled trial, quasi-experimental, longitudinal cohort, or case-control); a judgement was made if the comparison groups were comparable;

• evaluate an intervention that could be conducted by municipalities; and

• be published in English.

In addition, there is a need for a clarification of which interventions that might be included or excluded on the basis of the "could be conducted by municipalities" criterion, because municipalities are organized differently across the world. Accordingly, interventions including legislative reforms, interventions targeting workers, and interventions including modifications of vehicles have been excluded in this review. On the other hand, interventions targeting nursing home residents have been included. Furthermore, interventions do not need to have been undertaken by municipalities to be included; municipalities should, however, have the possibility to implement the intervention at hand.

### Exclusion criteria

A study was excluded if

• the intervention included any form of medication or drug use;

• it was assessed as not being relevant to the general context; and

• it had an unacceptable quality, appraised by using a checklist previously used by The Swedish Council on Technology Assessment in Health Care [7].

Review articles were excluded, although they were later revised to see if they would add any valuable information.

### Sifting process

Sifting was systematically conducted in three different steps and was carried out by one assessor. In the first sift titles and abstracts were screened; in the second sift papers were obtained and screened if they met the inclusion and exclusion criteria; in the third sift papers were critically appraised by using a checklist [7]. Only papers with at least an acceptable quality were included.

### Quality assessment

In the last sift, articles were critically appraised using a modified checklist for evaluating health economic studies [7]. The checklist is in turn based on Drummond's checklist [8]. The assessment of an article's quality cannot be given with mathematical precision, therefore The Swedish Council on Technology Assessment in Health Care propose three categories to express the quality of a study: High, acceptable, or not acceptable quality, which corresponds to more than 80, 50-80 or less than 50 percent positive answers, respectively, of the applicable checklist questions [7]. If an article had previously been reviewed, and was available on the Centre for Reviews and Dissemination's database, that review was used to validate the quality assessment. When there were ambiguities, a revision was conducted.

### Data extraction

Findings from the included studies were extracted into pre-developed evidence tables. To facilitate comparisons all cost estimates were converted to US dollars in price year 2007 by first using GDP deflators and then Purchasing Power Parities (PPPs) as suggested by the Campbell & Cochrane Economics Methods Group [9]. Apt data were retrieved from IMF and OECD [10,11]. If the price year was not reported in the article, the year before publication was used when converting cost data. Critical appraisals of the quality of the included studies were also conducted. Furthermore, the interpretation and validity was briefly discussed. In the case of ambiguities a more detailed perusal was carried out.

### Meta-analysis

Due to the research question and to the heterogeneity in economic evaluation methodologies it is neither feasible nor meaningful to conduct a meta-analysis [12].

## Results

### Result of the sifting process

**Figure 1:**
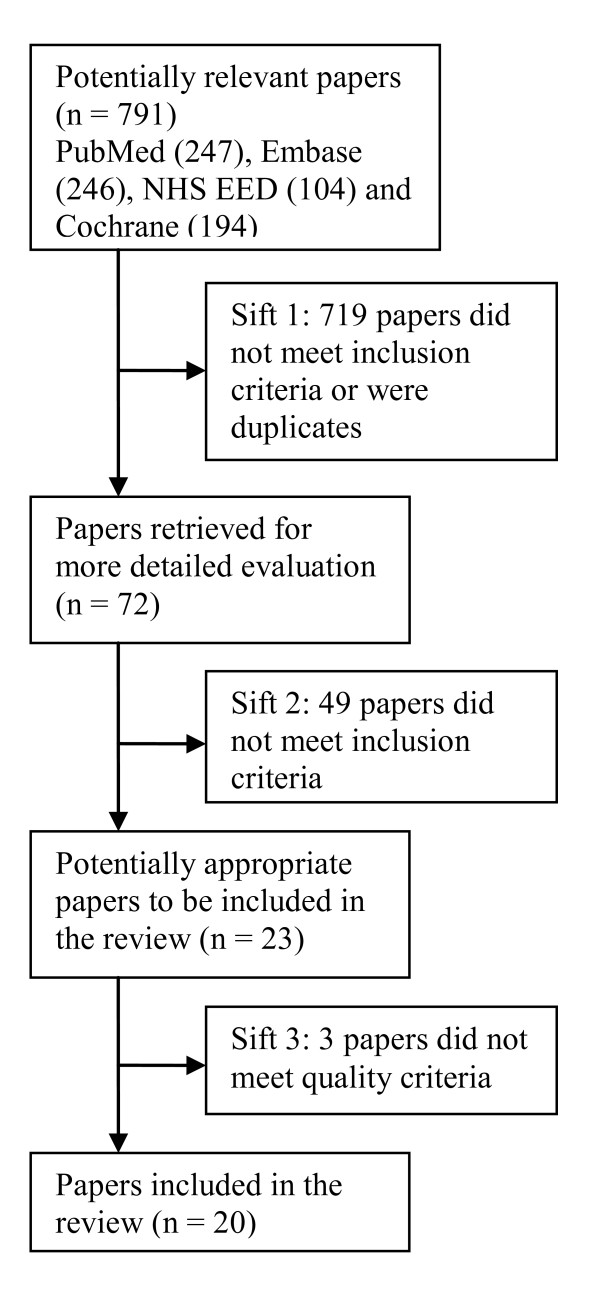
**Flow chart showing sifting process**.

The systematic literature search yielded 791 potentially relevant papers. Following a sifting process in three steps shown in Figure 1, 20 articles were finally included in the systematic review. Most articles were discarded due to violating the inclusion or exclusion criteria, although many duplicates were also excluded. In the final sift three articles, which did not meet the quality criterion, were excluded.

### Interventions by area, economic result, setting, and population

The included articles comprise different kinds of injury prevention interventions shown in Table 1 by area and by economic result. The table clearly shows that there are few articles in each area except in fall reduction and the use of hip protectors. Furthermore, many studies report a potential of being cost saving and only three studies report no effects.

**Table 1:** Papers reviewed by area and result. Numbers in parentheses show articles with effectiveness data generated within the study.

Area	Number of papers n = 20	Showing net savings	Showing a cost per health score gained	Showing no effects
Community-based, multitargeted	1	1		

Traffic safety	1	1^a^	1^a^	

Smoke alarm	2	1(1)		1

Fall reduction	6	2^b^	4(3)^b^	1

Hip Protectors	9	7^c^	4^c^	1(1)

Sports	1	1(1)^d^	1(1)^d^	

Total	20	14	10	3

^a ^One study reported both net savings (time horizon 8 years) and a cost per health score gained (time horizon 1 year) and is thus reported twice [31].

^b ^One study reported both net savings (time horizon 10 years) and a cost per health score gained (time horizon 1 year) and is thus reported twice [27].

^c ^Included are three articles that report both net savings and a cost per health score gained and are thus reported twice each. One study show net savings without nursing aide time added and a cost per health score gained if added [17]. Another study show net savings for an older population [15]. The third study show net savings for a high-risk population [14].

^d ^One study reported both net savings (time horizon "long term") and a cost per health score gained (time horizon 36 weeks) and is thus reported twice [33].

Studies were also conducted across different settings, compromising eight studies from North America, eight from Europe, and four from Australia and New Zeeland as well as different populations, with the main focus on older people with 15 interventions solely targeting them.

### Methodological characteristics and synthesis of the included studies

The extracted data from the included studies are delineated and juxtaposed in a table enclosed in Additional file 3. First out are six articles that base their findings on effectiveness data generated within the study, followed by 14 articles using effectiveness data reported elsewhere. The quality and validity of the included studies are appraised and briefly discussed qualitatively in the column furthest to the right in the table. In general, there are great methodological varieties between studies which make comparisons more difficult.

The limited number of studies in each area makes evidence difficult to synthesise in specific areas. However, nine studies examined the use of hip protectors [13-21]. Many reported cost saving results although one randomised controlled trial reported no significant effects [21].

Six studies targeted fall reduction in elderly people. One randomised controlled trial of a multidisciplinary program reported no effect [22]. Four studies calculated a cost per prevented fall and the sixth study reported a QALY (Quality Adjusted Life Year) gain and a minor saving [23-27].

Two studies examined smoke alarm give-away interventions in high risk areas [28,29]. One reported net-savings and one no effects. All other studies investigated interventions not directly comparable and are, hence, only reported in Table 1 and in Additional file 3.

## Discussion

### Principal findings

This systematic review identified few economic evaluation studies of injury prevention interventions, of acceptable quality, that might be conducted at the municipal level. Hence, it is shown that there is a paucity of economic evidence - especially in low- and middle-income countries - in this field. Consequently, the evidence based options, including economic information, available for a decision-maker at the municipal level are limited. However, the review reveals that many interventions included in the review are reported to be effective and also cost saving. This shows a potential for societies to obtain good value for societal resources spent.

In general, it seems like interventions targeted at high-risk individuals are more cost-effective. Probably, this is the case if the cost of identification of high-risk individuals does not reduce cost-effectiveness. Furthermore, the results indicate great disparities in methodologies used, which make comparisons more difficult. The outcomes of many interventions are also context dependent. Hence, the results and their transferability should be interpreted with caution.

### Interpretation of the included studies

Before interpreting the studies some caveats should be noted. Different interventions cannot directly be compared because there might be, for instance, contextual, compositional, or methodological differences. Especially, in economic evaluations, with disparities in methods, there are difficulties to synthesise evidence into a coherent whole [30]. Consequently, caution should be exercised when interpreting, synthesizing, and generalising results.

Some interpretations could however be made on the basis of the included studies. Many interventions including the use of hip protectors were reported as cost saving. However, the only study that did not import effectiveness data from other sources reported no significant effects [21]. This may be due to the low compliance reported and that the study was underpowered. Nevertheless, it is likely that compliance is a key factor for achieving effectiveness; and that cost-effectiveness will be enhanced if compliance could be improved. Moreover, some of the studies suggest that interventions targeting older groups are more cost-effective than interventions targeting younger groups. Some of the studies also suggest that women profit from the use of hip protectors at an earlier age than men do.

Among fall prevention interventions in elderly, most were reported as being effective. In four interventions the cost-effectiveness was reported as a cost per fall prevented. If we do not know the value of a prevented fall this information is of limited value for decision-makers. One study reported that the investigated program was very likely to be cost-effective and encourage similar programs [24]. On the other hand, another study found no significant effects of the program, and thus did not recommend it [22]. There are also contextual and compositional differences between interventions, which influence outcome and make comparisons and synthesis difficult. However, Salkeld et al. concludes that their program is likely to be more cost-effective among older people with a history of falls [26]. Hence, it may be the case that interventions targeted at high risk individuals could give more value for money than interventions targeted at medium and low risk individuals.

The evidence seems conflicting in smoke alarm give-away interventions in high risk areas. It seems that many smoke alarms stopped functioning [28,29]. Functioning smoke alarms is likely to be a crucial part of successful smoke alarm interventions, and if the life time of smoke alarms could be enhanced this might improve the outcome of similar programs. All remaining studies are too heterogeneous to synthesis, and are thus reported solely in the table in Additional file 3[21,31-33].

To conclude, analysing all the interventions, it seems that many interventions are targeted towards high-risk individuals and that some interventions show better cost-effectiveness for high-risk groups. It is plausible that interventions targeted towards high-risk individuals have a potential to be more cost-effective than interventions targeted to the general population as long as identifying high-risk individuals is relatively inexpensive.

### Strengths and weaknesses of this review

First, the review is systematic, including a comprehensive search strategy in several major databases. Hence, most of the relevant published studies should have been captured. Second, the quality and validity of the included studies are reviewed, which simplify the interpretations and conclusions of the studies. Third, the review provides a better decision basis by including economic evidence, when deciding about implementing injury prevention interventions. Fourth, the review guides future research by giving an overview of the existing economic evidence in injury prevention.

This study is not without limitations. First, only peer-reviewed literature in English has been considered. Accordingly there may be a risk of publication bias; that is, there is a risk of that negative findings might not have reached publication to the same extend as positive findings [34]. Possibly important non peer-reviewed literature could also have been omitted. Second, even though the assessor used established checklists in the sifting and quality assessment processes there are always elements of subjectivity. Also, using only one assessor enhances the risk of error. To minimise these risks a validation of the quality appraisal was conducted with the help from abstracts, written by health economists, in the databases of Centre for Reviews and Dissemination and Euronheed, when available; 15 out of 20 articles were found in these databases and the unanimity was high between the appraisals. Third, this review only included identified studies with an economic perspective - see Anderson [12] for a discussion about the limitations. This fact disregards evidence including only effectiveness data. To obtain better knowledge, it would have been desirable to conduct comprehensive systematic reviews on the health related effectiveness, besides collecting information about the costs, of each intervention of interest. That would however be more resource demanding. Fourth, economic models, which are included in this review, often rely on assumptions and data collected from various sources. This makes the results more prone to uncertainty and therefore the results should be interpreted with caution. Finally, the interventions included in the review do not give a fair picture of the populations and settings where the burden of injury is the greatest; thus the results are biased in the following ways: 1. The interventions are limited to high-income countries although 90% of injuries occur in low- and middle-income countries [35]. 2. Falls account for only 8% of all injuries, yet most of the included interventions relate to falls [35]. 3. The review is mainly focused on elderly even though almost 50% of the world's injury-related mortality occurs in people aged between 15-44 years [36]. 4. Suicide, homicide, drowning, and poisoning are causes that account for approximately 29% of all injury deaths [35]; despite this, no interventions targeting these causes have met the inclusion criteria.

### Comparison with previous reviews

To the author's knowledge this is the first systematic review with a health economic perspective in this area. Other reviews, with economic perspectives, have studied more specific topics. For instance, Smith and Fordham have reviewed the cost-effectiveness of "interventions in reducing the risk of new falls, or modifying the harm caused in the event of a fall" among elderly. One of their conclusions is that there are very few economic studies relating to fall prevention programs [4]. The scarcity of economic studies is in line with the conclusions in this study. Miller and Levy have made estimates from literature reviewed concerning cost-outcome analyses in injury prevention and control [5]. From 84 estimates made in the United States they conclude that injury prevention interventions often can reduce medical costs and save lives. This is also in line with the findings in this study.

## Conclusions

The findings of this systematic review can be concluded in three main points: injury prevention interventions can reduce injuries and costs at the same time, there is a paucity of evidence, and there is a need for more standardised research.

First, the findings of this systematic review suggest that some injury prevention interventions seem to offer good use of societal resources, and some that do not. This information of specific programs is valuable and useful to decision-makers deciding about their implementation. However, it is important to note that contextual factors often play a pivotal role for the outcome of interventions. Consequently the results from individual studies should be seen as indicative rather than precise when transferring them to other settings. Furthermore, it is vital to recognize that political and other noneconomic factors also influence decisions. Hence, economic evidence should be an important part in decision-making but there are other important parts as well.

Second, the review reveals a deficiency of economic evidence surrounding injury prevention interventions - especially in low- and middle-income countries - at the municipal level. This calls for further research. Further research should be targeted at areas where there is a lack of knowledge - as this review reveals - and where increased knowledge could be beneficial. See, for instance, Jonsson for a guide in selecting topics to assess used by The Swedish Council on Technology Assessment in Health Care SBU [37]. One possible way to go forward is also to construct economic models and populate them with the best available evidence. That is a relatively cheap way to obtain valuable information for decision-makers and one example of that is the article: Modeling the cost effectiveness of injury interventions in lower and middle income countries: opportunities and challenges by Bishai and Hyder [38].

Third, it is important that future economic evaluations use similar methodologies to simplify comparisons. For instance, if perspectives other then the societal are requested, then the societal perspective should also be included in the analysis. Increased knowledge and standardisation of evaluations facilitates decision-making; partly by increasing the options available for a decision-maker asking for economic evidence and partly by facilitating comparisons.

Furthermore, it would be valuable to explore the marginal effects (that is, dose-response relationships) of different injury prevention interventions to find the optimal level. To get this knowledge we need a "more detailed understanding of *how *different combinations and levels of resources, and their associated opportunity costs, *cause *different patterns of outcomes" in different populations and contexts [12]. Hence, it is important for future research to target these issues.

## Competing interests

The author declares that they have no competing interests.

## Supplementary Material

Additional file 1 **Search strategy overview**. The file shows an overview over the search strategies employed, for the different databases. Click here for file

Additional file 2 **Search strategies**. Click here for file

Additional file 3 **Description of the included articles. Studies with imported effectiveness data and model studies are presented last**. Click here for file
