# Supplementary material for: Cost-effectiveness of injury prevention - a systematic review of municipality based interventions
Source: Cost Eff Resour Alloc. 2010 Sep 10;8:17. doi: 10.1186/1478-7547-8-17 (PMC2945985; doi:10.1186/1478-7547-8-17)
Supplement: Additional file 1 — Search strategy overview. The file shows an overview over the search strategies employed, for the different databases. [file 1478-7547-8-17-S1.DOCX]

| **PubMed** |  |  |  |  |  |  |
| --- | --- | --- | --- | --- | --- | --- |
| Accident prevention[MeSH] | |  | AND | Cost-Benefit Analysis[MeSH] | NOT | historical-article[pt] |
| OR |  |  |  |  |  | OR |
| Wounds and injuries[MeSH] | AND | prevention and control[Subheading] |  |  |  | editorial[]pt |
|  |  | OR |  |  |  | OR |
|  |  | primary prevention[MeSH] |  |  |  | letter[pt] |
|  |  | OR |  |  |  |  |
|  |  | Protective devices[MeSH] |  |  |  |  |
|  |  |  |  |  |  |  |

MeSH terms were limited to: published in the last 10 years. Additional searches were conducted to include the not yet MeSH indexed articles. The entry terms of the MeSH terms were used for searches limited to the preceding 180 days.

| **Embase** |  |  |  |  | | |
| --- | --- | --- | --- | --- | --- | --- |
| accident prevention'/exp | |  | AND | cost effectiveness analysis'/exp | AND | humans |
| OR |  |  |  | OR |  |  |
| injury'/exp | AND | protective equipment'/exp |  | 'cost utility analysis'/exp |  |  |
|  |  | OR |  | OR |  |  |
|  |  | primary prevention'/exp |  | 'cost benefit analysis'/exp |  |  |
|  |  | OR |  |  |  |  |
|  |  | protection'/exp |  |  |  |  |
|  |  |  |  |  |  |  |

Searches were limited to the period between 1998 and 2008

| **NHS EED** |  |  |  |  |
| --- | --- | --- | --- | --- |
| MeSH Accident Prevention |  |  | AND | MeSH Cost-Benefit Analysis |
| OR |  |  |  |  |
| MeSH Wounds and Injuries | AND | MeSH Primary Prevention |  |  |
|  |  | OR |  |  |
|  |  | MeSH Protective Devices |  |  |
|  |  |  |  |  |

Searches using MeSH terms were limited to the period between 1998 and 2008. Additional searches were conducted to include the not yet MeSH indexed articles. The entry terms of the MeSH terms were used for searches limited to the year 2008.

| **Cochrane** |  |  |  |  |
| --- | --- | --- | --- | --- |
| MeSH descriptor Accident Prevention | | | AND | MeSH descriptor Cost-Benefit Analysis |
| OR |  |  |  |  |
| MeSH descriptor Wounds and Injuries | AND | MeSH descriptor Protective Devices |  |  |
|  |  | OR |  |  |
|  |  | MeSH descriptor Primary Prevention |  |  |
|  |  |  |  |  |

Searches using MeSH terms were limited to the period between 1998 and 2008. Additional searches were conducted to include the not yet MeSH indexed articles. The entry terms of the MeSH terms were used for searches limited to the year 2008.
