# Supplementary material for: Cost-effectiveness of injury prevention - a systematic review of municipality based interventions
Source: Cost Eff Resour Alloc. 2010 Sep 10;8:17. doi: 10.1186/1478-7547-8-17 (PMC2945985; doi:10.1186/1478-7547-8-17)
Supplement: Additional file 2 — Search strategies. [file 1478-7547-8-17-S2.DOCX]

**Embase**

#1 'protective equipment'/exp

#2 cost effectiveness analysis'/exp OR 'cost utility analysis'/exp OR 'cost benefit analysis'/exp AND [humans]/lim AND [1998-2008]/py

#3 'injury'/exp

#4 'accident prevention'/exp

#5 'primary prevention'/exp

#6 'protection'/exp

#7 #1 OR #5 OR #6

#8 #3 AND #7

#9 #4 OR #8

#10 #2 AND #9 AND [humans]/lim AND [embase]/lim AND [1998-2008]/py

**NHS EED**

#1 MeSH Accident Prevention EXPLODE 1

#2 MeSH Protective Devices EXPLODE 1 2 RESTRICT YR 1998 2008

#3 MeSH Wounds and Injuries EXPLODE 1 RESTRICT YR 1998 2008

#4 MeSH Cost-Benefit Analysis EXPLODE 1

#5 MeSH Cost-Benefit Analysis EXPLODE 1 RESTRICT YR 1998 2008

#6 MeSH Primary Prevention EXPLODE 1 2 RESTRICT YR 1998 2008

#7 #1 AND #4 RESTRICT YR 1998 2008

#8 #3 AND #6 AND #5 RESTRICT YR 1998 2008

#9 #3 AND #2 AND #5 RESTRICT YR 1998 2008

#10 #7 OR #8 OR #9

#11 Analyses, AND Cost-Benefit OR Analysis, AND Cost-Benefit OR Cost-Benefit AND Analyses OR Cost AND Benefit AND Analysis OR Analyses, AND Cost AND Benefit OR Analysis, AND Cost AND Benefit OR Cost AND Benefit AND Analyses OR Cost AND Effectiveness OR Effectiveness, AND Cost OR Cost-Benefit AND Data OR Cost AND Benefit AND Data OR Data, AND Cost-Benefit OR Cost AND Benefit OR Benefits AND Costs OR Costs AND Benefits OR Cost AND Utility RESTRICT YR 1998 2008

#12 Prevention, AND Accident OR Accident AND Preventions OR Preventions, AND Accident RESTRICT YR 2008 2008

#13 #11 AND #12 RESTRICT YR 2008 2008

#14 #10 OR #13

**PubMed**

#1 Search Search Prevention, Accident OR Accident Preventions OR Preventions, Accident Limits: published in the last 10 years

#2 Search Device, Protective OR Devices, Protective OR Protective Device OR Safety Devices OR Device, Safety OR Devices, Safety OR Safety Device Limits: published in the last 10 years

#3 Search Search Wounds, Injury OR Trauma OR Traumas OR Wounds and Injury OR Injury and Wounds OR Injuries and Wounds OR Injuries, Wounds OR Wounds OR Wound OR Injuries OR Injury Limits: published in the last 10 years

#4 Search Analyses, Cost-Benefit OR Analysis, Cost-Benefit OR Cost-Benefit Analyses OR Cost Benefit AnalysisOR Analyses, Cost Benefit OR Analysis, Cost Benefit OR Cost Benefit Analyses OR Cost Effectiveness OR Effectiveness, Cost OR Cost-Benefit Data OR Cost Benefit Data OR Data, Cost-Benefit OR Cost Benefit OR Benefits and Costs OR Costs and Benefits OR Cost Utility Limits: published in the last 10 years

#5 Search "Primary Prevention"[Mesh] Limits: published in the last 10 years

#6 Search "Cost-Benefit Analysis"[Mesh] Limits: published in the last 10 years

#7 Search "Accident Prevention/economics"[Mesh] Limits: published in the last 10 years

#8 Search #7 AND #6 Limits: published in the last 10 years

#9 Search "Wounds and Injuries/economics"[Mesh] Limits: published in the last 10 years

#10 Search "prevention and control "[Subheading] Limits: published in the last 10 years

#11 Search "Protective Devices"[Mesh] Limits: published in the last 10 years

#12 Search "Wounds and Injuries"[Mesh] Limits: published in the last 10 years

#13 Search #12 AND #11 AND #6 Limits: published in the last 10 years

#14 Search #12 AND #5 AND #6 Limits: published in the last 10 years

#15 Search #9 AND #10 AND #6 Limits: published in the last 10 years

#16 Search #15 OR #14 OR #13 Limits: published in the last 10 years

#17 Search #16 OR #8 Limits: published in the last 10 years

#18 Search historical-article[pt] OR editorial[pt] OR letter[pt] Limits: published in the last 10 years

#19 Search #17 NOT #18 Limits: published in the last 10 years

#20 Search Prevention, Primary Limits: published in the last 10 years

#21 Search preventive therapy OR prophylaxis OR preventive measures OR prevention OR control Limits: published in the last 180 days

#22 Search #3 AND #21 AND #4 Limits: published in the last 180 days

#23 Search #3 AND #20 AND #4 Limits: published in the last 180 days

#24 Search #3 AND #2 AND #4 Limits: published in the last 180 days

#25 Search #22 OR #23 OR #24 Limits: published in the last 180 days

#26 Search #1 AND #4 Limits: published in the last 180 days

#27 Search (#25 OR #26) NOT #18 Limits: published in the last 180 days

#28 Search #27 OR #19 Limits: published in the last 10 years

**Cochrane**

#1 MeSH descriptor **Accident Prevention** explode all trees

#2 MeSH descriptor **Cost-Benefit Analysis** explode all trees

#3 MeSH descriptor **Protective Devices** explode all trees

#4 MeSH descriptor **Primary Prevention** explode all trees

#5 (#1 AND #2), from 1998 to 2008

#6 MeSH descriptor **Wounds and Injuries** explode all trees

#7 (#6 AND #3 AND #2), from 1998 to 2008

#8 (#6 AND #4 AND #2)

#9 (#5 OR #7 OR #8), from 1998 to 2008

#10 "cost utility"

#11 "cost benefit"

#12 "cost effectiveness"

#13 wound* OR injur*

#14 device* AND (protective OR safety)

#15 accident

#16 prevent*

#17 (#10 OR #11 OR #12)

#18 (**#13** OR **#15**)

#19 (#14 OR #16)

#20 (#17 AND #18 AND 19)

#21 (#20), in 2008

#22 (#9 OR #21)
