# Supplementary material for: Cost-effectiveness of injury prevention - a systematic review of municipality based interventions
Source: Cost Eff Resour Alloc. 2010 Sep 10;8:17. doi: 10.1186/1478-7547-8-17 (PMC2945985; doi:10.1186/1478-7547-8-17)
Supplement: Additional file 3 — Description of the included articles. Studies with imported effectiveness data and model studies are presented last. [file 1478-7547-8-17-S3.DOC]

## Description of the included articles. Studies with imported effectiveness data and model studies are presented last.

| **Author, year, country & design** | **Intervention** | **Study population**  **& setting** | **Perspective,**  **comparator,** **time horizon & discount rate** | **Costs**  **included** | **Effects, resources saved & drop-out** | **Cost-effectiveness^a^** | **Study quality and authors’ conclusions** |  |
| --- | --- | --- | --- | --- | --- | --- | --- | --- |
| **Haddix [29], 2001, US, CG and pre and post comparisons, CEA.** | Smoke alarm give-away int. Alarms and educational material were distributed to 10,100 households. The int. reached about78% of the households. | Homes without a functioning smoke alarm. High risk area for residential fire injuries, Oklahoma city. | Societal. Do nothing. Int. costs had a 5 year horizon. Lifetime for production loss.  3% for costs. | Int. and medical costs and production losses due to fire related injuries. | In comparison with the rest of the city the net change was -6.08, - 67.04 and -92.7% in fires, fatalities and non-fatal related injuries, respectively. | Net savings exceeded $22 millions. The results were robust to small changes in variable values. Thus, the intervention dominated no intervention. | The study design entails a risk for other factors to influence the result, although the risk should be small. CE would be improved if QoL would have been included in the analysis. Production losses were included and estimated to $1,123,124 per fatality and $11,450 per non-fatal injury. Only 58% of the participating households had a functional smoke alarm four years after the intervention. The authors argue that similar programs should be considered for implementation in high risk areas. |  |
| **Campbell [23], 2005, NZ, RCT, partial CEA.** | To reduce injuries and falls a home safety asse-ssment and modification int. (n = 100), a home exercise int. plus vita-min D (n = 97) or both int. (n = 98) were imple-mented. CG (n = 96) | People aged 75+ with low vision. Mean age ≈ 84. Living in the community in Dunedin and Auckland, New Zealand. | Societal stated.  Social visits.  1 year.  N/A. | Intervention costs | Only the home safety int. reduced falls sig. at 0.59 (CI: 0.42-0.83). No resources saved were estimated. In the exercise int. only 18% adhered fully. | When adjusted to fall events per 100 person years the total cost of implementing the program per fall was $796. That value ranged between $563 and $1,922 in the sensitivity analysis. | An RCT provides good evidence if properly conducted. This study showed that a home based safety assessment and modification program reduced the incidence of falls in the investigated pop. at a certain cost. However, medical and indirect costs, and QoL measurements, were not included, and we don’t know how much a prevented fall is worth. Thus, comparisons are complicated. The authors conclude that pro-grammes should be targeted at particular pop. groups and that low levels of adherence possibly caused the no effect result in the exercise int. |  |
| **Robertson [25], 2001, NZ, RCT, CEA.** | Home based muscle strengthening and balance int. Individually prescribed exercise that comprised (30 min exercise + 30 min walking) * 3/week. IG (n=116); CG (n=117). | Women aged 80+ (mean age ≈ 84) living in the community; invited by their GP from 17 general practices in NZ. | Societal.  Usual care including social visits.  1-2 years.  No discount rate. | Int., health care, and over head costs. | RR was 0.68 (0.61) for falls (injurious falls). Sig. better score in the SF-36 physical measure. No sig. differences in costs. 71% agreed to continue. | The ICER was $265 ($224) in year 1(year 2) per fall prevented. Per fall resulting in a moderate or serious injury it was $386 ($360). | An RCT provides good evidence if properly conducted. One can question if social visits is a comparable alternative to usual care. Too small sample size to demonstrated sig. in cost difference. Limited usefulness of the results until we know the value of a prevented fall. Longer evaluation period may improve CE. If indirect costs and QoL measures would have been valued and included then it is likely that the CE would improve and it would facilitate comparisons. The authors recommend that an exercise program designed to reduce falls for older people should be considered. |  |
| **Salkeld [26],**  **2000, Aus, randomised trial, CEA.** | Home hazard reduction program to reduce falls. Visit (1 h) by an occupational therapist followed by home modifications. IG (n=264); CG (n=266). | 65+, living in the community, recruited when visiting health care. Mean age ≈ 77 year. | Societal. Routine care after discharge.  1 year. Equipment was annuitized and discounted at 5%. | Int., hospital, other institutional health care, home health care, and informal care costs. | RR was 0.64 in the subgroup with a fall history but not overall (p=0.07). No sig. differences in costs and physical and mental status (SF-36) were shown between the groups. | Average (median) costs were $4,761 ($2,725) for all subjects and $3,801 ($114) for the high risk group per fall prevented. The IG used more health care. The int. was cost sav-ing for the high risk group after removing outliers. | A bigger sample would have increased the power. There were differences in non-hospital and informal care mean costs between the groups prior to int. start ($3,810 for IG and $2,884 for CG), which may influence the results. It appears that most health care costs were unrelated to falls, and the authors conclude that the increase in health care costs is a chance result. This makes it difficult to ascertain the true costs related to falls. If indirect costs and QoL measures would have been valued and included then it is likely that the CE would improve. The authors conclude that the int. is likely to be most CE among older people with a history of falls. |  |
| **van Schoor [21], 2004, NL, RCT, CEA.** | HPs were randomised individually. 4-5 HPs per individual. IG (n=276); CG (n=285). | Frail institutionalized elderly, 70+, mean age ≈ 86 year. | Societal  Not wearing the protector.  1 year. N/A | Int. and direct health care costs. | No sig. effects. No sig. cost differences. Compliance was 37% after 1 year. | Due to no sig. effects there was no CE reported. | The study seemed underpowered. Also, compliance was poor. Costs seemed well described although no indirect costs and QoL measures have been included. Tytex provided the HPs. Funded by grants from VAZ Doelmatigheid and Praeventiefonds. |  |
| **Verhagen [33], 2005, NL, RCT, CEA.** | Balance board training during warm up for less than 5 minutes. 36 week long. IG (n=641); CG (n=486). | Dutch volleyball players, second and third division. Mean age ≈ 24. | Societal.  Comparator was usual training without balance board.  36 week follow-up.  N/A. | Int., direct health care, indirect (both unpaid and paid work [friction cost approach]) costs.  Cost diaries were used and unit prices obtained. | The incidence of ankle sprain was sig. lower for the IG compared to CG: 0.5 and 0.9, respectively, per 1,000 playing hours. Costs were sig. higher for the IG. | Preventing one ankle sprain costs ≈ $572 ($67) for all pop. (previously injured players). If int. costs were distributed over the long term, the int. would be cost beneficial. | An RCT should provide good evidence. Only 40% of the invited teams participated, which make the study prone to selection bias, and only 54-59% completed cost diaries. For paid work there were 63% higher costs in the IG. Questionable if all int. costs should be included. No time costs were calculated for the int. The local cost estimates and the limited sensitivity analysis makes generalisation difficult. QoLwas not included. The authors conclude that positive effects were achieved at certain costs. The int. can be recommended if it is prolonged. |  |
| **Studies with imported effectiveness data and model studies** | | | | | | | | |
| **Ginelly [28], 2005, UK, cluster RCT, CEA. Econometric model.** | Smoke alarm give-away int. with information. Wards (40) were randomised to IG or CG. 20,050 smoke alarms were distributed. | Material deprivation wards, London. Smoke alarm prevalences were 47% (wards) and 72% (national). | Societal. Wards were pair matched according to material deprivation. 2 year follow-up. | Int., fire service, police, property damage, and health service. Unit cost data used. | Higher probability of having a fire in the IG. The difference was sig. and RR was 1.116. No sig. differences in costs. | A CE acceptability curve was depicted. When WTP was 0 then there was an 18% probability for the int. to be CE. The probability decreased with an increase in WTP. | Difficult to assess the quality of the estimated effectiveness because it was reported elsewhere. Costs seemed appropriate calculated and were taken from various sources. Production loss and QoL were not included. Few alarms were distributed: 20,000 in an area of 73,400 homes (27%). A trial showed that almost half of the installed alarms did not work after 15 months. Context dependent due to high risk area. The authors write that a similar int. is unlikely to represent a CE use of societal resources. |  |
| **Lindqvist [32], 2001, SWE, based on a quasi-experimental, CBA .** | A community-based injury prevention int. consisting of traffic, sports and recreation, and workplace safety int. with environmental and behavioural parts. | The int. was focused on elderly, teenagers and children in Motala (a WHO Safe Community with pop. 41,000). | Societal.  Control municipalities. | Int., injuries, time value, and production loss. | Injuries (non- trivial injuries) decreased by 13% (41%). Health care costs amounted to 23% of the total cost. | The net savings were ≈ $1.3 million. If prolonged and a longer time perspective is used then the annual net savings amount to 2.6 million. | Effects were taken from previous published literature. The IG had a higher incidence level from the start and that difference ceased to exist after the int. Incidence rates were recorded during shorter periods which may influence the results due to the stochastic nature of injuries. Costs seemed correctly estimated. The value of a reduced number of injuries was collected from one study. QoL is not included in the analysis; if included the CE would be enhanced. The authors judged the int. as CE. |  |
| **Goldstein [31], 2008, US, CEA, decision tree.** | Child Restraint System (CRS) disbursement and education. A convertible seat for children between 0-4 year and a belt positioning booster seat between 4-8 years. | Hypothetical cohort of 100,000 low-income children. Enrolment at birth and 8 year cycle,  USA. | Societal,  No int.  1-8 year time horizon  3%. | Int., direct and indirect medical costs, future productivity loss, and parental work loss. | The int. was assumed to increase proper CRS (booster seat) use by 23% and 35% for infants/toddlers and children aged 4-7 years, respectively. | The costs ($) per death averted (life year gained) were 4,442,481 (133,927) and -104,364 (-3,146) for 1 and 8 years perspective. The results were sensitive to changes in variables. | It is difficult to appraise the quality of the estimates used in the study because they were collected from various sources. Hence, the results should be interpreted with caution. In the study over 80% of the crash-related costs are future productivity loss. The authors conclude that implementing a similar int. is an important potential strategy for addressing injury disparities among low-income children. Funding from State Farm Mutual Automobile Insurance Company. |  |
| **Johansson [24], 2008, SWE, quasi-experimental, CUA,**  **Markov model.** | Community-based elderly safety promotion int. that target acciden-tal falls. Five year int. combining environmen-tal structural changes with individually based measures. | Urban community, 18% aged 65+, ≈ 5,500; and among those 1,400 were 80+. | Societal. No int. in several control areas. 5 year int. costs. Savings and benefits up to age 100 if alive.  3%. | Int., medical care, community care, and informal care costs. Collected prospectively. | No RR stated. They estimate that 14 hip-fractures were avoided during a six year period and that 35 QALYs were gained at a net saving of $8,200. | A small saving close to zero and a gain of 35 QALYs. The do nothing alternative was thus dominated. Around 1 of 25 hip fractures could potentially be avoided by the program. | The study design was quasi-experimental and effectiveness was determined by a time series analysis. Int. costs were collected prospectively which enhances validity. Fracture related costs and QoL changes were taken from other sources; thus it is difficult to assess the validity of those figures. In an extensive sensitivity analysis the cost per QALY varied between <0 and $26,500. The authors conclude that the int. is very likely to be CE and encourage similar int. The data used are solely Swedish which make generalisations to other settings complicated. |  |
| **Hendriks [22], 2008, NL, RCT, CEA & CUA.** | Multidisciplinary fall prevention program including medical and occupational therapy assessments by a geriatrician and an OT, respectively. IG (n=166); CG (n=167). | Community-dwelling elderly people (65+) were recruited if they visited a hospital due to a fall. Mean age ≈ 75 year. | Societal.  Usual care.  1 year.  N/A. | Int., health care, patient and family. Standardized cost prices were used. All health care costs were included; not only fall related costs. | No sig. effects in number of people sustaining a fall. Follow-up was 1 year. QALYs were measured with EQ-5D. 25% drop-out in IG after 1 year. | The analysis showed no effect of the int. and consequently no CE. Sensitivity analysis did not change conclusions. | Based on an RCT described elsewhere. A disadvantage is that no information was given about how effective the assessments were in changing medications, behaviours, and environments etc., which are crucial parts. Longer follow-up period could also alter the results. All health care costs were included, which make it difficult to ascertain the difference due to falls between the groups. No leisure time costs were included. The int. was cost neutral but the authors did not recommend it in its present form in the NL. |  |
| **Smith [27], 1998, Au, CEA, decision-tree model.** | Home assessment and modification int. to reduce falls. An OT did the assessment and then followed a provision of fall-prevention devices. About 4 h was needed. | People aged 75+ living independently in the community. | Health service viewpoint. 1 and 10 year horizon.  5%. | Int. including equipment, hospital, some rehabilitation, and home help costs. | RR was assumed to be 0.75. The fall (injury after fall) rate was assumed to be 0.4 (0.1).  Drop-out wasn’t addressed. | The ICERs ($ per fall [injury] prevented) were 1,666 [16,662] and -887 [-8,866], respectively, for the 1 and 10 year horizon.  Results are sensitive to variations in key variables. | The effectiveness was taken from 1 study and it wasn’t described how it was found. Costs were mainly local and collected from different sources and were assumed in some cases. No indirect costs and no QoL were included; only intermediate variables, i.e. number of falls and injurious falls. No leisure time costs were included. Only one-way sensitivity analyses were performed. The authors conclude that the results are indicative and that further research is required to provide firm data. |  |
| **Sawka [17], 2007, Canada, CEA.** | Provision of HPs. 3 pairs per year. HP price of $79 | Nursing home residents aged 65+ (N=60,775, 51% age 85+, female/male rate ≈ 3/1). Ontario. | Ontario ministry of health (third-party-payer). Not wearing HPs. 1 year horizon. No discount rate. | Acute HF costs (hospitalisation, physicians and over head costs). HF costs were estimated to $10,395. No extra time costs for staff were assumed in the base-case scenario. | RR was 0.29. Annual incidence of HF was assumed to be 4.3%. The mean number of avoided HFs per year was estimated to 1,864. Compliance was assumed to be similar to other studies and was reflected in the RR. | The mean yearly cost savings per HF prevented was estimated to $2,998 (CI: -13,136-19,838). The probability of being cost saving was 0.64. If 5 min nursing aide time is added the cost saving is changed to -$10,144 per HF preven-ted with a probability of 0.11 of being cost saving. | The effectiveness of HPs is taken from a meta-analysis which is described elsewhere. That makes it difficult to judge the validity. The perspective was not societal which makes comparisons with other interventions more difficult. For instance, it is shown that the CE ratio is very sensitive to variations in variables, especially if nursing aide time is added. No indirect costs were assumed. QoL is not included in the analysis. The analysis is also context dependent. All in all, it is difficult to make any conclusions about the results although the authors report that there is a reasonable probability that such a strategy may result in health care savings. |  |
| **Honkanen [15], 2006, US, CUA, state-transition Markov Model with 10,000 simulations.** | HP use. 4-7 and 3-5 pairs per year were assumed to be used by nursing home residents and community members, respectively.  Individuals transitioned between yearlong health states. | Hypothetical cohort of individuals aged 65 and older without a previous HF and initially living at home. Community and institutional care. | Societal but used medical costs to third-party payers as a proxy for all medical costs.  Do nothing.  Lifetime horizon. 3% | Medicare costs up to 12 months after fracture. | RRs for fractures were 0.52 (0.16 for efficacy). QALY loss was 0.312 (0.01) due to fracture (discom-fort of wearing HPs). Drop-out was 0.5-0.63 but fell in subsequent years. | HP use saved costs and gained QALYs in women (men) initiating use at age 80 (85). In women age 75, the ICER was $21,000/ QALY and in men age 80 HP saved costs but decreased QALYs. | The efficacy rate used is derived from 1 criticized study which decreases the validity. The costs were Medicare costs and not societal, which complicate comparisons. Patients out-of-the pocket, time, and production loss were not included. QALY decrements due to fractures were taken from 1 study. The distributions of the variables weren’t reported. The authors conclude that HP use save costs and QALYs for older age cohorts of both sexes, and that additional research on QoL effects of wearing HPs is warranted. |  |
| **Meyer [16], 2005, Germany, based on a cluster RCT, CEA.** | HP use and education (60 min) with written material for nurses and residents. 3 HPs for 18 months. IG 25 clusters (n = 459); CG 24 clusters (n = 483). | Nursing home residents aged 70+. Mean age was 87.  Hamburg. | Health and nur-sing care insur-ance viewpoint. The CG got 2 HPs and brief information. 18 month horizon. | Int., medical, and nursing care costs after HFs. | The risk reduction was estimated to 4.1% in other publications. No sig. difference in mean HF related care costs. | The ICER reported in the base case scenario was $1,444 per additional HF avoided. In the sensitivity analysis the ICER varied between $514 and $1,981. | Generally, an RCT provides good evidence but it was described elsewhere which make it difficult to appraise the validity. The risk reduction (4.1%) seems modest. The costs seem to be properly collected, although some costs are missing from a societal perspective. QoL was not addressed. Compliance, which is crucial, was not investigated. The authors conclude that the program studied deserves consideration of implementation for its low incremental costs per additional HF avoided. |  |
| **Fleurence [14], 2004, UK, CUA, Markov model.** | HP use. On average 1 HP/year. Males and females were divided in two groups: a high risk group (previously incurred a fracture) and others. | Four hypothetical cohorts of 1,000 individuals in each. Males and females (70 years old). Primary, secondary, and residential care. | National Health Service (NHS)  No treatment.  Lifetime.  6%. | Direct costs to the NHS. Fracture costs included were acute, social care, nonacute, and drug costs. Treating cost for one HF was estimated to $22,640. | RR for HP was 0.25. Compliance was set to 35%. Utilities were obtained for different ages. A multiplier of 0.797 was used to determine the QoL the year after HF and subsequent years. | ICERs ($/QALY) were: for females (males) 13,716 (55,493) for the general pop. and cost saving (19,912) for a high-risk pop., in a lifetime perspective. | Effectiveness data was taken from a systematic review. The compliance rate ranged between 20% and 92% (median 56%) in the literature and was set to 35%. The assumption about the long term reduction in QoL after HF enhances CE and is questionable due to uncertainties around the long term effects. The discomfort of wearing HPs is not reflected. Furthermore, assumptions had to be made when data weren’t available. Mainly UK data were used which limits generalisations to other settings. The author concludes that at a ceiling ratio of $20,000/QALY, HPs are CE in the general female pop., in the high-risk male pop., and cost-saving in the high-risk female pop. A fee was received from Eli Lilly. |  |
| **Singh [19], 2004, Canada, CEA & CUA, decision analytic model.** | HP use to prevent hip fractures. 3 pairs per year. | Hypothetical cohort of 1,000 nursing home residents similar to a real pop. in White Rock, BC.  Mean age was 85. | Societal.  No treatment. 12 month horizon for risks and costs and lifetime for QALYs. 3%. | Direct medical, int. costs. No long-term rehabilitation costs. Hospital treatment of HF was estimated to $15,800. | RR was 0.37 (0.24-0.56). Compliance is reflected in the RR. QoL was assumed to be 0.63 and reduced by 0.2 (0.1) in the first year (subsequent years), after fracture. | The costs ($) for HPs: -10,419 per HF prevented and -15,759 (-17,770) per QALY gained for female (male). Negative figures show cost-savings. The results were robust to changes in variables. | Effectiveness data were derived from a Cochrane review. The utilities were collected from one study with a younger age group; this influences the results. The discomfort of using HPs wasn’t addressed. No indirect and longer-term rehabilitation costs were included; if so, that would probably enhance CE. Compliance wasn’t addressed in the sensitivity analysis, although reflected in the RR. The authors conclude that the CEA suggests that HPs could save money while preventing HF and improving QoL in nursing home residents. |  |
| **Colón-Emeric [13], 2003, US, CUA, decision tree model.** | HP use. 3 pairs per year. | High-risk elderly.  Nursing home facilities. Durham. | Societal with a time horizon of 18 months.  Do-nothing alternative.  3%. | Medical, HPs.  No extra labour time costs in the base-case scenario. | RR was 0.46 for HPs. After a HF the QoL decreased from 0.48 to 0.28 and was assumed to be constant over time. | The use of HPs saves ≈ $360 per subject and adds 0.01 QALYs over 18 months in the base-case scenario. Sensitivity analysis did not change the main results. | Effectiveness data were not derived by a systematic review, and compliance was not reflected in a sensitivity analysis. QoL after HF was taken from 1 study and was assumed to be constant. No ages were reported for the included individuals, which limits generalisation. Costs were taken from published literature and a cost to charge ratio of 0.58 was used. The authors conclude that the use of HPs in nursing facilities (high-risk patients) is cost saving or economically attractive. |  |
| **Waldegger [20], 2003, Canada, CUA, decision tree model.** | HP use. 7 pairs per year. | A women aged 82 with a previous fracture. Institutional dwelling elderly. | Perspective not stated.  Do-nothing alternative.  Time horizon was unclear but seems to be two years. 5%. | HPs, fracture costs up to 1 year after fracture.  Cost of HF was $19,359 (price year was not stated but if 2000). | RR was 0.4 for HPs. For a HF the QoL decreased from 0.92 to 0.4 (0.5), for the first (second) year after fracture. Compliance was 25-50%. | The use of HP saved costs and gained QALYs compared to not wearing HPs. | The effectiveness data was collected through a meta-analysis and seemed to have been properly conducted. Wrist and vertebral fractures are included in the analysis, and one can question if they are related to wearing HPs. The fracture costs and utilities were each collected from 1 study. No indirect costs included. QoL was 0.92, which seems high for a woman at age 82. Furthermore, the loss in QoL seems very high compared to other studies which enhance CE. The authors conclude that there is evidence to support the use of HP for the studied pop. |  |
| **Segui-Gomez [18], 2002, US, CUA, state-transitional model.** | HP use. 1 pair per year. | Two hypothetical cohorts of 500,000 65+ men or women.  Community in USA. | Societal,  No HPs.  Lifetime or up to 100 years of age.  3%. | Net medical costs associated with averted mortality and morbidity, HPs.  Cost of fracture was $45,321. | RR was estimated to 0.44. QoL losses were 0.14 for HF and 0.005 for HP use. Compliance was varied in a sensitivity analysis. | Base case results show that HP use result in net cost savings for elderly women (men) over age 65 (75). Women over age 65 and men over age 85 also gain QALYs through HP use. | Effectiveness was taken from a large RCT. The loss in QoL after a HF was assumed to be constant until death and that is likely to be an overestimation. The cost analysis seemed properly conducted although no indirect costs were included. The discomfort of using HPs was estimated. Data has been collected from various sources. Hence, there are uncertainties surrounding the results. The authors conclude that their analysis indicates that the use of HPs is associated with cost savings. |  |

^a^ All cost estimates have been converted to US dollars in price year 2007.

CBA = Cost Benefit Analysis, CE = Cost-Effective(ness), CEA = Cost-Effectiveness Analysis, CG = Control Group, CUA = Cost Utility Analysis, HF = Hip Fracture, HP = Hip Protector, ICER = Incremental Cost-Effectiveness Ratio, Int. = Intervention, IG = Intervention Group, N/A = Not Applicable, OT = Occupational Therapist, Pop. = Population, QoL = Quality of Life, RCT = Randomised Controlled Trial, RR = Relative Risk, Sig. = Significant (α = 0.05), WTP = Willingness To Pay, ≈ = approximately.
